# Interspinous Process Decompression Improves Quality of Life in Patients with Lumbar Spinal Stenosis

**DOI:** 10.1155/2018/1035954

**Published:** 2018-07-02

**Authors:** Pierce D. Nunley, Vikas V. Patel, Douglas G. Orndorff, William F. Lavelle, Jon E. Block, Fred H. Geisler

**Affiliations:** ^1^Spine Institute of Louisiana, Shreveport, LA, USA; ^2^The Spine Center, University of Colorado Hospital, Denver, CO, USA; ^3^Spine Colorado, Mercy Regional Hospital, Durango, CO, USA; ^4^Upstate Bone and Joint Center, East Syracuse, NY, USA; ^5^San Francisco, CA, USA; ^6^Chicago, IL, USA

## Abstract

Lumbar spinal stenosis has been shown to negatively impact health-related quality of life. Interspinous process decompression (IPD) is a minimally invasive procedure that utilizes a stand-alone spacer to serve as a joint extension blocker to relieve neural compression in patients with spinal stenosis. Using the 5-year results from an FDA randomized controlled trial of IPD, the quality of life in 189 patients treated with the Superion® spacer was evaluated with the SF-12. Physical and mental component summary (PCS, MCS) scores were computed preoperatively and at annual intervals. For the PCS, mean scores improved from 29.4 ± 8.1 preoperatively to 41.2 ± 12.4 at 2 years (40%) and to 43.8 ± 11.6 at 5 years (49%) (p<0.001 for both comparisons). At 2 years, 81% (103 of 128) of subjects demonstrated maintenance or improvement in PCS scores. The mean MCS score improved from 50.0 ± 12.7 preoperatively to 54.4 ± 10.6 and 54.7 ± 8.6 at 2 and 5 years, respectively (p>0.10 for both comparisons). These results demonstrate that the significant impairment in physical well-being found in patients with lumbar spinal stenosis can be ameliorated, in large part, by IPD treatment.

## 1. Introduction

The classic clinical feature of lumbar spinal stenosis is neurogenic claudication which causes intermittent lower extremity pain and diminished functional abilities [1]. As symptoms progressively grow more chronic and refractory to conservative measures, spinal stenosis inevitably leads to systemic impairment of overall well-being and quality of life [2].

Interspinous process decompression (IPD) devices (aka “spacers”) were developed to address a distinct therapeutic gap in the continuum of care of lumbar spinal stenosis treatment [3]. Interspinous spacers effectively bridge the often lengthy interval between failed conservative care and the point where surgical decompression becomes necessary to manage intractable symptoms. These devices are inserted posteriorly via a minimally invasive procedure without disruption of osseous or ligamentous tissues. Spacers provide immediate symptom amelioration by serving as a spinal extension blocker to prevent the repetitive compression of neural elements during back extension that is the primary source of claudicant symptoms.

The Superion spacer is the only stand-alone IPD device available on the US market. There is an extensive body of published research supporting the safety and effectiveness of this device for treatment of moderate lumbar spinal stenosis [4–9]. Importantly, IPD has also been shown to offer similar symptom relief as operative decompressive laminectomy [10, 11]. Herein, we described the results of long-term IPD treatment on generic health-related quality of life.

## 2. Materials and Methods

Health-related quality of life outcomes through 5 years of postoperative follow-up were obtained from the Superion (Vertiflex, Carlsbad, CA USA) treatment arm of a randomized controlled FDA noninferiority trial comparing two interspinous spacers. This multicenter trial evaluated the use of stand-alone IPD in the treatment of subjects aged 45 or older with moderate symptoms of intermittent neurogenic claudication, secondary to a diagnosis of moderate degenerative lumbar spinal stenosis at one or two contiguous levels from L1 to L5. Three hundred ninety-one subjects met the trial eligibility criteria and were randomized to treatment. The Superion was approved by the FDA in 2015 for commercial distribution based on the 2-year primary endpoint analysis [8]. Additionally, the condition-specific clinical outcomes have been reported through 5 years of follow-up [5–7]. The current quality of life analysis was restricted exclusively to the Superion arm of the trial.

This investigational device exemption (IDE) trial complied with all US regulatory requirements and was approved by the Institutional Review Board at each participating site, and patients provided written informed consent before any study-related procedures were performed. The trial was prospectively registered at ClinicalTrials.gov (NCT00692276).

As assessment of health-related quality of life was included as an ancillary clinical measurement in this trial. It was not included as component of the primary study composite endpoint or as a secondary condition-specific outcome such as the Zurich Claudication Questionnaire (ZCQ), leg and back pain severity by visual analog scale, or the Oswestry Disability Index (ODI). Prior to surgery as well as at annual intervals, quality of life was evaluated in study subjects using the SF-12® Health Survey, version 2 (SF-12) [12]. The SF-12 is a 12-item questionnaire used to assess patient-reported generic health outcomes, such as general health and well-being, including the impact of any and all illnesses on a broad range of functional domains. The SF-12 consists of a subset of 12 items from the SF-36® Health Survey (SF-36) covering the same eight domains of health outcomes, including physical functioning, role-physical, bodily pain, general health, vitality, social functioning, role emotional, and mental health [13]. The SF-12 provides two composite scores: the physical component summary (PCS) and the mental component summary (MCS). These summary scores are computed using the scores of the twelve questions and range from 0 to 100, where zero indicates the lowest level of health and 100 indicates the highest level of health. Based on a general US population sample, both the PCS and MCS scores are linearly transformed to adjust scores to a mean of 50 and standard deviation of 10 [14].

The percentage improvement in PCS and MCS at the 2-year primary trial endpoint and the 5-year follow-up interval compared to preoperative values was computed and the results were displayed graphically. The degree of improvement was analyzed using the paired t-test, 2-tailed. We also computed the percentage of subjects that maintained or improved in PCS and MCS score over baseline at the same follow-up intervals.

Of 189 patients initially randomized to Superion treatment, SF-12 questionnaire responses were captured in 144, 128, 96, 55, and 68 study subjects at 1, 2, 3, 4, and 5 years, respectively.

## 3. Results

**Figure 1:**
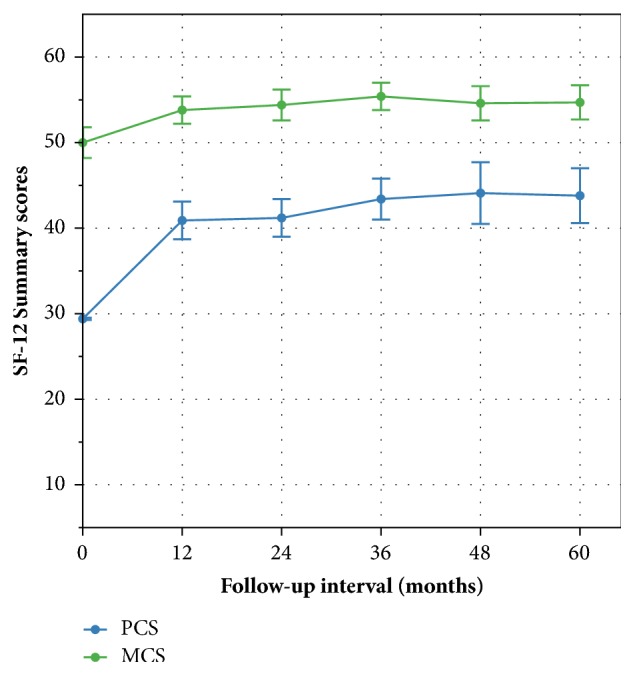
Time course of results for the physical component summary (PCS) and mental component summary (MCS) scores through 5 years of follow-up. Results presented as mean (95% CIs).

Longitudinal changes in PCS and MCS scores through 5 years of follow-up are illustrated in Figure 1. The mean PCS score improved from 29.4 ± 8.1 preoperatively to 41.2 ± 12.4 at the 2-year primary trial endpoint and to 43.8 ± 11.6 at 5 years, representing average percentage improvements of 40% and 49%, respectively (p<0.001 for both comparisons). At 2 years, 81% (103 of 128) of subjects demonstrated maintenance or improvement in PCS scores. Similarly, 87% (59 of 68) of subjects who provided 5-year SF-12 responses continued to maintain or improve their PCS score.

The mean MCS score improved from 50.0 ± 12.7 preoperatively to 54.4 ± 10.6 and 54.7 ± 8.6 at 2 and 5 years, respectively, representing a 9% improvement at both follow-up intervals (p>0.10 for both comparisons). At 2 and 5 years, 60% (77 of 128) and 57% (39 of 68) of subjects showed maintenance or improvement in PCS scores.

## 4. Discussion

IPD treatment has been shown to provide durable condition-specific symptom amelioration through 5 years of follow-up [6]. The current findings extend these previous results and demonstrate significant improvement in overall well-being following IPD, particularly in the physical function domains of quality of life.

There has been a limited number of previous reports of the association between IPD and quality of life outcomes. Using a first-generation IPD spacer compared with conservative care in a randomized trial, Hsu et al. [15] evaluated quality of life using the SF-36 questionnaire instrument in patients with moderate lumbar spinal stenosis. At all posttreatment time points, mean SF-36 domain scores in IPD-treated patients were significantly greater than those in patients treated conservatively, with the exception of the mean general health (GH), role emotional, and MCS scores at 2 years. Additionally, mean posttreatment domain scores in IPD-treated patients were significantly greater than mean pretreatment scores, with the exception of mean GH scores. The results of this study demonstrated that IPD was significantly more effective than conservative care in improving the quality of life in patients with lumbar spinal stenosis.

Previously reported feasibility studies with the Superion spacer reported strikingly similar results to the current study in terms of improvements realized in physical functioning. In a pre-IDE study, Bini et al. [16] reported on a total of 121 patients with moderate lumbar spinal stenosis treated with IPD and followed for 12 months. Using the SF-36 questionnaire instrument, these authors reported a percentage improvement of 41% over preoperative scores for the PCS. Also employing the SF-36, Shabat et al. [17] found a PCS improvement of 40% in 53 IPD-treated patients with intermittent neurogenic claudication followed for 2 years.

Preoperatively, we noted significant impairment in the PCS scores of patients enrolled in this FDA-IDE trial, with an average value (29.4) that was more than 2 standard deviations below normal. Conversely, the mean preoperative MCS score (50.0) was identical to the US norm-based average, showing no impairment. These findings corroborate results reported from a large community-based sample of patients with lumbar spinal stenosis evaluated with the SF-36 [2]. In this observational study of 1,862 individuals, the presence of symptomatic lumbar spinal stenosis had a strong negative impact on the PCS, but not the MCS.

In a randomized trial comparing laminectomy with laminectomy plus fusion, Ghogawala et al. [18] also noted depressed preoperative PCS scores in patients with lumbar spinal stenosis (e.g., 34.7, laminectomy; 31.5, laminectomy/fusion). At 2 years postoperatively, percentage improvements in PCS were 27% and 48% for laminectomy and laminectomy plus fusion, respectively.

We demonstrated that IPD treatment improves PCS scores by > 40% through 5 years of postoperative follow-up. Importantly, mean posttreatment PCS scores increased to and remained consistently in the low-normal range, within one standard deviation of the US norm.

Because the IPD implantation procedure is performed in a minimally invasive fashion and causes only minor anatomical disruption, the full range of surgical options remains available if a revision becomes necessary to manage reemergence of symptoms. Thus, with simplicity of the operative procedure, rapid patient recovery, low surgical risk of complications, and long-term clinical durability, IPD remains a viable treatment option for stenosis patients.

## 5. Conclusions

In addition to its documented benefits on condition-specific efficacy outcomes, interspinous process decompression with a stand-alone spacer also offers significant improvement in overall health-related quality of life, particularly in areas of physical functioning.
